# Epidemiologic and Genomic Reidentification of Yaws, Liberia

**DOI:** 10.3201/eid2704.204442

**Published:** 2021-04

**Authors:** Joseph W.S. Timothy, Mathew A. Beale, Emerson Rogers, Zeela Zaizay, Katherine E. Halliday, Tarnue Mulbah, Romeo K. Giddings, Stephen L. Walker, Nicholas R. Thomson, Karsor K. Kollie, Rachel L. Pullan, Michael Marks

**Affiliations:** London School of Hygiene and Tropical Medicine, London, UK (J.W.S. Timothy, K.E. Halliday, S.L. Walker, N.R. Thomson, R.L. Pullan, M. Marks);; Wellcome Sanger Institute, Hinxton, UK (M.A. Beale, N.R. Thomson);; Ministry of Health, Monrovia, Liberia (E. Rogers, Z. Zaizay, T. Mulbah, R.K. Giddings, K.K. Kollie);; University College London Hospitals NHS Foundation Trust, London (S.L. Walker, M. Marks)

**Keywords:** yaws, Treponema pallidum, pertenue, neglected tropical diseases, Liberia, West Africa, case finding, case detection, accessibility, genome, sequencing, nonhuman primate, bottleneck, bacteria

## Abstract

We confirmed endemicity and autochthonous transmission of yaws in Liberia after a population-based, community-led burden estimation (56,825 participants). Serologically confirmed yaws was rare and focal at population level (24 cases; 2.6 [95% CI 1.4–3.9] cases/10,000 population) with similar clinical epidemiology to other endemic countries in West Africa. Unsupervised classification of spatially referenced case finding data indicated that yaws was more likely to occur in hard-to-reach communities; healthcare-seeking was low among communities, and clinical awareness of yaws was low among healthcare workers. We recovered whole bacterial genomes from 12 cases and describe a monophyletic clade of *Treponema pallidum* subspecies *pertenue*, phylogenetically distinct from known TPE lineages, including those affecting neighboring nonhuman primate populations (Taï Forest, Côte d’Ivoire). Yaws is endemic in Liberia but exhibits low focal population prevalence with evidence of a historical genetic bottleneck and subsequent local expansion. Reporting gaps appear attributable to challenging epidemiology and low disease awareness.

Yaws, caused by the bacterium *Treponema pallidum* subspecies *pertenue* (TPE), is an infection of skin and soft tissues, affecting primarily children, with transmission driven by direct human-to-human contact (*1*). TPE disseminates through the bloodstream and lymphatic system and can lead to extensive ulcerative or papillomatous lesions and progressive damage to cartilage or bone. In eradication campaigns that ran during 1948–1964, >300 million persons were assessed for yaws and >50 million were treated with injectable benzathine penicillin, reducing global prevalence by as much as 95% (*2*). Despite this achievement, interest in yaws eradication waned and the disease resurged in several countries in Africa, the Pacific, and Southeast Asia by the 1970s. In 2012 the World Health Organization (WHO) relaunched eradication efforts based on total community and targeted treatment with single-dose azithromycin, termed the Morges strategy (*3*).

Historically, 103 countries have reported cases of yaws, but as of 2018 only 14 continued to report confirmed cases to WHO (*4*). It remains unclear whether this reflects true absence of disease or rather inadequate surveillance and loss of disease-specific expertise (*5*). A recent modeling study suggested that more than two thirds of countries without recent data would be highly unlikely to report yaws without dedicated active surveillance (*4*). Furthermore, since the launch of the Morges strategy, the Philippines remains the only country that previously reported cases to subsequently confirm autochthonous transmission (*6*). Surveillance activities are challenging because of low population-level prevalence with cases clustered (*7*,*8*) among poor rural populations with low accessibility (*9*), although there is a lack of objective data on yaws-endemic communities. Consequently, no standardized approaches exist to efficiently identify cases in areas of unknown burden (*10*,*11*). One approach proposed by WHO to is to integrate active surveillance for multiple neglected tropical diseases (NTDs) that affect the skin (skin NTDs) (*12*), including yaws, an approach recently adopted by several countries in West Africa (*13*).

For *T. pallidum* subspecies *pallidum*, genetic epidemiology has informed understanding on global transmission patterns and macrolide resistance (*14*,*15*). Despite their close genetic relationship, few whole-genome sequences are currently available for TPE. Furthermore, yaws-like disease caused by TPE has now been detected in nonhuman primate species in several countries in sub-Saharan Africa, including Côte d’Ivoire (*16*,*17*). The direct role of nonhuman primate species as a potential reservoir for zoonotic infection is currently unknown, but it may prove critical to guiding eradication strategies (*16*). Yaws eradication efforts will be supported through improved genomic analyses of human and nonhuman primate TPE at different spatiotemporal scales to inform understanding of transmission and antimicrobial resistance.

Yaws was previously highly endemic to Liberia; active clinical prevalence was estimated to be as high as 30% during the first eradication era (*18*). National surveillance systems, however, ceased reporting cases by the mid-1970s, and no yaws cases have been confirmed in subsequent decades (*5*,*19*). Several countries in the region do continue to report high numbers of yaws cases (*5*), including neighboring Côte d’Ivoire, and there have been anecdotal reports of unconfirmed cases in Liberia in recent years. As part of an integrated project on skin NTDs, we undertook an exhaustive population-based burden estimation in Maryland County, Liberia, and identified 24 cases of serologically confirmed yaws. We present detailed epidemiologic and whole-genome characterization of these cases and their affected communities.

## Methods

### Setting and Survey Design

We conducted a population-based cross-sectional integrated survey for skin NTDs (Buruli ulcer, leprosy, lymphatic filariasis–associated morbidity, and yaws) during June–October 2018 in Maryland County, Liberia (census population 165,456) (Appendix 1). Maryland County is in the far southeast part of Liberia and borders known yaws-endemic regions in Côte d’Ivoire. All communities and residents were eligible for inclusion. We defined community health worker catchment areas as primary sampling units (PSUs), stratified them across all 24 health facilities, and systematically selected them using probability proportional to size. The study protocol was approved by the University of Liberia (PIRE) Institutional Review Board (no. 18-02-088) and the Ethics Committee of the London School of Hygiene and Tropical Medicine (no. 14698).

### Procedures

Community health workers undertook exhaustive screening for skin NTDs by visiting all households, door to door, in their selected PSUs. The community health workers showed photographs of common skin NTD lesions to household members who verbally reported whether they, or other household members, currently exhibited skin lesions similar to those in the photographs. Suspected skin lesions identified by community health workers were subsequently verified by specially trained nonphysician healthcare workers during follow-up surveys. For the purpose of this survey, we focused on the typical lesions of primary yaws, namely ulcerative lesions and papillomas. All persons with lesions compatible with yaws or tropical ulcer, and any child (<18 years of age) with any form of ulcer were tested using a rapid treponemal test (SD Bioline, https://www.globalpointofcare.abbott); if that result was positive, then they were tested with the syphilis dual path platform (DPP) lateral flow assay (ChemBio, https://chembio.com). During clinical diagnosis, details of healthcare-seeking behaviors and previous diagnoses were recorded, then confirmed at health facilities. For clinically suspicious cases of yaws or any other ulcers, swab specimens were collected from the largest lesion. Lesion samples were shipped to the London School of Hygiene and Tropical Medicine, where a multiplex quantitative PCR (qPCR) assay for both TPE (targeting *polA*) and *Haemophilus ducreyi* (*hhdA*), and a separate qPCR for *Mycobacterium ulcerans*, the causative agent of Buruli ulcer (*IS2404*), were performed. All serologically confirmed yaws cases (DPP positive) were referred for immediate treatment with azithromycin.

### Outcome

We defined yaws infection as being positive for both treponemal and nontreponemal antibodies on testing with the DPP assay. We defined active yaws as a clinically suspicious lesion in which TPE was detected by qPCR from a lesion sample. Survey activities also included measurement of other skin NTDs (Appendix 1).

### Data Analysis

We estimated prevalence through design-based inference as a stratified 1-stage cluster design with variance estimated using jackknife repeated replication (R version 4.0.1, survey version 3.36; https://www.r-project.org). The intraclass correlation coefficient (ICC) of PSUs was estimated from intercept-only binomial mixed effects models (lme4 version 1.1–23) (*20*). We extracted community accessibility data from all household GPS point locations (n = 9,375) collected by community health workers during screening from open-source GIS layers (raster version 3.1–5). Locations with missing data (n = 489) following extraction were removed and median values aggregated by PSU for analysis. We used unsupervised classification to objectively define subpopulations of PSUs with lowest accessibility in Maryland County. We classified PSUs using nested (K-means) or partitioning (hierarchical agglomerative and divisive) clustering methods (cluster version 2.1.0). We used Euclidean distance as a standard measure and limited cluster number between 2 and 5. We applied Ward’s method for hierarchical agglomerative classification. We chose the optimal approach using weighted rank aggregation across 3 measures of internal validity: Dunn index, silhouette width, and adjusted connectivity (optCluster version 1.3.0) (*21*) leading to selection of divisive hierarchical classification for final analysis.

### Whole-Genome Sequencing

From all TPE PCR-positive swabs in the study, we submitted samples with a qPCR threshold of <Cq 32 for whole-genome sequencing using the pooled sequence capture method described previously (*14*,*22*). We mapped *Treponema*-specific sequencing reads to the Samoa D reference genome (bwa mem version 0.7.15), as previously described (*15*), to infer a whole genome multiple sequence alignment (samtools version 1.6, bcftools version 1.6, http://www.htslib.org), contextualized by 33 high-quality publicly available TPE genomes (and 1 *T. pallidum* subsp. *endemicum* as outgroup) from around the world. We used Gubbins version 2.4.1 (https://github.com/sanger-pathogens/gubbins) to mask recombination and generated a maximum likelihood phylogeny (IQ-Tree version 1.6.10, http://www.iqtree.org). Macrolide resistance-associated alleles were inferred as previously described (*15*). We inferred pairwise single nucleotide polymorphism distances between samples using pairsnp (https://github.com/gtonkinhill/pairsnp). We performed joint ancestral reconstruction using pyjar (https://github.com/simonrharris/pyjar).

## Results

**Figure 1:**
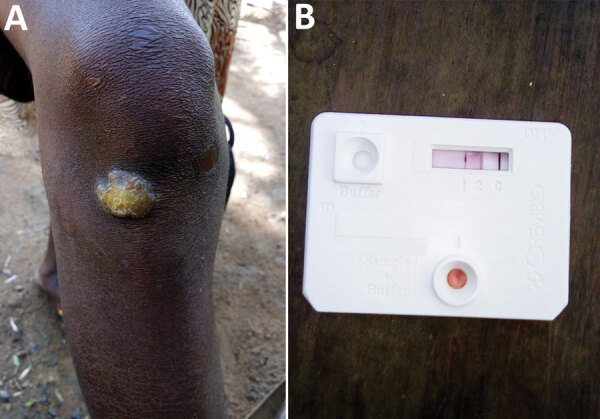
Clinical presentation and serological results of the first confirmed case of yaws since the 1970s and first whole *Treponema pallidum* subspecies *pertenue* (TPE) genome from Liberia. A) Papillomatous yaws lesion below the right knee. B) Paired serological results from this case. Dual path platform syphilis lateral flow assay (ChemBio, https://chembio.com) shows antibody binding to treponemal and nontreponemal antigen indicative of active yaws infection. A complete genome sequence was recovered from this case (Figure 3).

**Figure 2:**
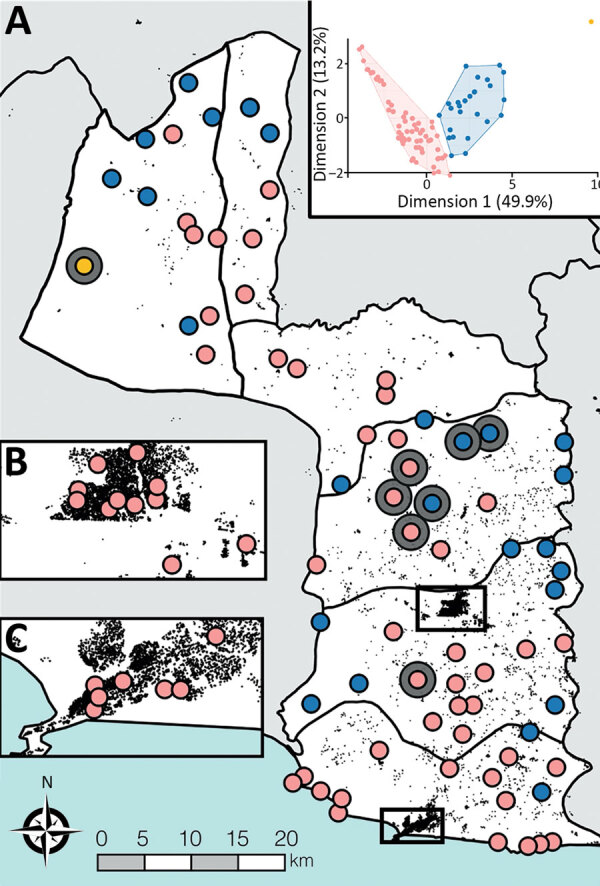
Spatial occurrence and accessibility of yaws endemic communities in Maryland County, Liberia. A) All survey cluster centroids (n = 92). Yaws endemic clusters are shown by large gray circles. All survey clusters were classified based on accessibility criteria into high access (pink), low access (blue), and very low access (yellow) using open-source GIS datasets. Black features are OpenStreetMap defined buildings (©OSM Contributors) to provide indication of structural density. B, C) Main urban centers of Maryland County: Pleebo (B) and Harper (C). Inset: Results of divisive hierarchical classification. The axes of this plot show the principal components and proportion of variance explained by each component.

After exhaustive screening of 56,825 persons from 92 clusters in Maryland County, we assessed 81 persons with ulcerative or papillomatous lesions meeting testing criteria for yaws, using an SD Bioline rapid treponemal test. We identified 28 treponemal seropositive persons who were subsequently tested using the ChemBio DPP. Among this group, we identified 24 cases of serologically confirmed yaws infection; of these, 17 were PCR-confirmed active yaws lesions. We estimated the design-adjusted population prevalence of serologically-confirmed yaws infection as 2.6 (95% CI 1.4–3.9) cases/10,000 population (Table). Of note, the first case of yaws was not confirmed until 36,621 persons had been screened (Figure 1), emphasizing the sampling effort in confirmation of the first case. The spatial distribution of cases was highly focal, with occurrence in only 8/92 (8.7%) survey clusters and an intraclass correlation coefficient estimated at 0.93 (Figure 2). Maryland County is divided into 6 health districts; confirmed cases were identified in 3 districts, including a single, isolated case within the most populated district, Pleebo (Figure 2). Spatial clustering was also evident from the concentration of 15 cases (62.5%) among clusters served by a single health facility, where the maximum cluster-level prevalence of 2.0% was observed.

**Table:** Descriptive epidemiological characteristics of serologically confirmed yaws cases, Liberia*

Characteristic	Value
Total serologically confirmed cases	24
Total PCR-positive lesions	17 (70.8)
Whole genome recovered	12 (50.0)
Prevalence of serologically confirmed yaws, cases/10,000 population (95% CI)	
Crude prevalence in survey population	4.2 (2.5–5.9)
Design-adjusted population prevalence	2.6 (1.4–3.9)
Clinical diagnostic accuracy, % (95% CI)	
Positive predictive value of all suspected yaws	64.7 (46.5–80.3)
Positive predictive value of yaws ulcers	47.6 (25.7–70.2)
Negative predictive value of tropical ulcers	94.4 (81.3–99.3)
Positive predictive value of yaws papilloma	92.3 (64.0–99.8)
Case demographics	
Median age, y(interquartile range)†	10 (8.2–12.0)
Sex*	
M	17 (73.9)
F	6 (26.1)
Clinical presentation	
Ulcer	12 (50.0)
Papilloma	12 (50.0)
No. active lesions‡	
1	17 (73.9)
2	3 (13.0)
3	2 (8.7)
10	1 (4.4)
Patient-reported duration of lesion	
<8 wk	9 (37.5)
8–26 wk	6 (25.0)
27 wk–1 y	5 (20.8)
1–3 y	0
>3 y	3 (12.5)
Unknown	1 (4.2)
Sought formal healthcare for lesion(s)	
Yes	11 (45.8)
No	13
Provided with any diagnosis from health provider	
Yes	1 (4.2)
No	23
Treated lesion(s) with prescription pharmaceuticals	
Yes	6 (25.0)
No	18
Sought traditional medicine for lesion(s)	
Yes	2 (8.3)
No	22

*Values are no. (%) except as indicated. †Missing data for 2 cases. ‡Missing data for 1 case.

Aside from 1 case in a 32-year-old person, all confirmed cases were in persons <18 years of age, most of whom were male (Table). The most common clinical presentation was a solitary skin lesion. The morphology of the skin lesions was either papillomatous (12/24; 50.0%) or ulcerated (12/24; 50.0%). A total of 11/12 (91.7%) papillomas had a positive PCR for TPE (a specimen was missing for 1 sample), compared with 6/12 (50%) yaws-like ulcers that had a positive PCR for TPE. In addition, 12 persons identified with clinically suspicious yaws lesions tested negative by yaws serology. Among this group, we detected *H. ducreyi* in 3 persons and *IS2404* from *M. ulcerans* in 2 persons. Most persons with yaws cases (15/24; 62.5%) reported having symptoms for <6 months, although 3 reported persistence of symptoms for >3 years.

Active healthcare seeking for treatment of yaws among users of the Maryland health system appeared low, as did clinical awareness among providers. Fewer than half of persons with confirmed cases reported seeking formal healthcare for lesions before survey activities (Table). Among those seeking care (n = 11), only 1 person received any formal diagnosis (tropical ulcer) although 6 (54.5%) received prescription pharmaceuticals. Use of traditional medicine providers was rarely reported for persons with confirmed yaws (2/24, 8.3% of cases) despite the common use of these pathways for other skin NTDs reported in our project (data not shown)*.* Only 1/7 (14.3%) nonphysician healthcare workers recruited for validation survey activities reported prior awareness of the clinical diagnosis of yaws during presurvey knowledge assessments. Despite this lack of awareness, the positive predictive value (PPV) of confirmed yaws among clinically suspicious cases was promising after a tailored training program for healthcare workers (64.5%, 95% CI 46.5%–80.3%). When broken down by major clinical forms, yaws papillomas demonstrated notably higher clinical PPV than ulcers (Table). Clinical teams were also trained to differentiate between multiple forms of ulcers. The negative predictive value (NPV) among all ulcers clinically diagnosed with non-yaws etiology was high (98.0%, 95% CI 93.1%–99.8%). NPV remained high (94.4%, 95% CI 81.3%–99.3%) when limited to only tropical ulcers, the major differential diagnosis of yaws ulcers.

**Figure 3:**
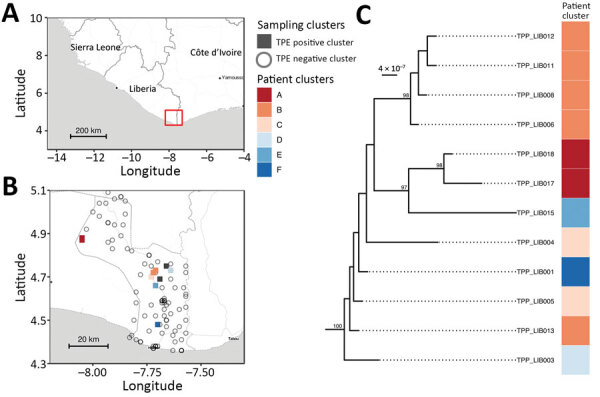
Spatial and phylogenetic distribution of 12 whole genome TPE sequences isolated from serologically confirmed yaws cases in Maryland County, Liberia. Genomes are extremely closely related but show evidence of geographical separation. A) Regional map with study area highlighted in red. B) Maryland County, indicating sampling location of *Treponema* genome (colored by survey cluster). C) Maximum-likelihood whole genome phylogeny of Liberia genomes, scaled by substitutions per site, showing phylogenetic relationships of patient samples. Ultra-fast bootstrap values >95% are indicated on the tree. Map tiles by Stamen Design (CC-BY 3.0), map data by OpenStreetMap (ODbl). TPE, *Treponema pallidum* subspecies *pertenue*.

To objectively classify the accessibility of survey communities in Maryland County, we used a divisive hierarchical classification algorithm that defined 3 distinct population groups, which we described as high accessible (65 clusters), low accessible (26 clusters), and very low accessible (1 cluster; Appendix 1). Yaws cases were identified in all 3 population groups (Figure 2); the proportion of yaws-endemic communities was inversely correlated with accessibility (high access: 3/65, 4.6%; low access: 3/26, 11.5%; very low access: 1/1, 100%; p = 0.041). Four cases of active yaws were identified in the single very–low-access cluster (Figure 3, cluster A, prevalence 1.3%). This community exhibited similar population density to low-access clusters, but estimated travel times to cities, calculated using friction surface data, were 2.6 times higher and estimated travel times to health facilities were 3.6 times higher. More than half the confirmed cases (14/24; 58.3%) were identified in high-access clusters, including 1 community with 10 cases and prevalence of 2.0% (Figure 3, cluster B). Among the high-access communities, no cases were identified in clusters a priori defined as urban or periurban.

We conducted a sensitivity analysis using alternative clustering algorithms (k-means and agglomerative hierarchical). Both outputs failed to define a very–low-access group, yet delineated low-access populations with strong concordance to the original grouping. Yaws cases were similarly distributed across these 2 groups but lacked statistical evidence of a difference (agglomerative; high, 4/66, 6.1%; low, 4/26, 15.4%; p = 0.22). We also repeated classification analysis with leprosy-endemic communities (Appendix 1). This showed that leprosy did not follow the same patterns as yaws, with cases most frequent in high-access clusters (22/65, 33.8%; low access, 5/26, 19.2%; very low access, 0/1; p = 0.38).

**Figure 4:**
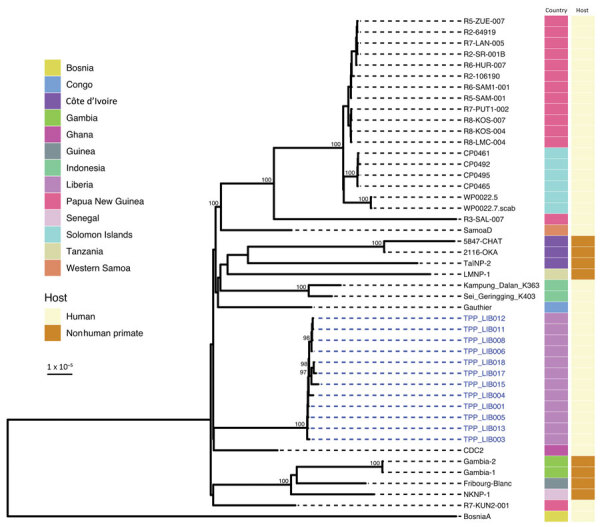
Global context of whole *Treponema pallidum* subspecies *pertenue* (TPE) genomes from Liberia. Liberia genomes form a monophyletic clade, genetically distant from publicly available genomes including 3 isolated from nonhuman primates in nearby Taï National Park (Côte d’Ivoire). Plot shows a maximum-likelihood phylogeny of 12 Liberia genomes contextualized with 34 published global genomes, scaled by substitutions per site. Ultra-fast bootstrap values >95% are indicated on the tree. Colored tracks show country of sampling and original host organism. Novel Liberia genomes from this study are indicated with blue labels.

We were able to recover whole genomes from 12 of the 17 PCR-positive lesion samples (Appendix 1 Table 1). We identified a novel monophyletic TPE clade in Maryland County (Figure 3) in which all sequences were phylogenetically distinct relative to publicly available TPE genomes isolated from humans and nonhuman primates (Figure 4). This distinction includes clear separation from the geographically related TPE genomes isolated in nonhuman primates from nearby Taï National Park (Côte d’Ivoire). All Liberia genomes were extremely closely related; 10 variable genome positions described the entire Liberia subtree, and a maximum of 7 pairwise single-nucleotide polymorphisms exist between any 2 TPE genomes. Ancestral reconstruction on the phylogeny inferred that the maximum number of substitutions from the common ancestor of all genomes sequenced from Maryland County was 4. Contextualized by previous estimates that the molecular clock rate of *T. pallidum* is 4–9 years/substitutions/genome (*14*,*23*), which suggests a recent common ancestor within the past 16–36 years. Despite low overall diversity, there remained evidence of local geographic separation within Maryland County genomes, particularly among 2 northernmost communities (Figure 3, clusters A, B). All sequences were predicted to be azithromycin sensitive based on in silico analysis of the 2 known azithromycin resistance loci (A2058G, A2059G) in the 23S ribosomal regions previously reported for syphilis or yaws.

## Discussion

We have demonstrated through deployment of comprehensive serologic and molecular tools that yaws remains endemic to Liberia and have provided detailed epidemiologic description of the cases and the affected communities. Our results also provide novel insight into the genetic epidemiology of yaws in West Africa and the operational challenges of identifying yaws cases in countries with unknown disease distribution.

The clinical profile of yaws in Liberia is similar to those from other endemic countries in West Africa: the disease is predominantly detected in male children who have a high proportion of papillomatous lesions (*24*). We showed that TPE genomes in Maryland County form a monophyletic clade that is clearly distinct from other publicly available TPE genomes. The low genomic diversity within samples from Liberia is consistent with previous observations regarding low mutation rates of TPE and TPA (*14*,*23*) yet contrasts the genetic and geographic structuring observed in the broader global phylogeny. This finding suggests evolution and expansion of local TPE strains from a common ancestor subsequent to the benzathine penicillin-based eradication campaigns conducted until the 1960s, rather than recent importation from elsewhere. This information contrasts with a recent study on highly endemic Lihir Island, Papua New Guinea, where TPE was polyphyletic and exhibited >3 distinct phylogenetic clades (*15*). These observations were made despite a smaller geographic area and population and may reflect both higher TPE transmission rates and mobility among island inhabitants. Repeated events similar to those observed in Liberia occurring across the West Africa region may also explain the broader geographic structuring (Figure 4). Under this scenario, circulating TPE populations may have undergone historic collapse as a consequence of previous eradication efforts, leading to genetic bottlenecks and subsequent expansion of isolated residual cases. Characterization of genomes from other spatially contiguous locations will, however, be required to better understand fine-scale transmission in this region.

The bacterial genomes we describe were recovered from human patients with yaws who were <200 km from Taï National Park and represent the closest geospatial overlap with nonhuman primate TPE genomes currently available (*16*). These human-derived TPE genomes appearing completely unrelated to those from nonhuman primates, coupled with our detection of only a single monophyletic clade, highlight the importance of geography on TPE population structure. This finding is also inconsistent with recent zoonotic transmission between humans and the nonhuman primates in this area (*17*), although more intensive, localized sampling is needed to affirm our understanding of TPE as a potential zoonosis.

Recent implementation of azithromycin mass drug administration (MDA) in Papua New Guinea led to the emergence of azithromycin-resistant strains of TPE (*25*). We found no evidence of azithromycin-resistant alleles in any TPE genomes in Liberia. To our knowledge, these genomes represent the western limit of human TPE genomes described in Africa and suggest low prevalence or selection pressure for resistance alleles in Maryland. The absence of azithromycin MDA programs across Liberia (*26*) for either yaws or trachoma is a key consideration for low selection pressure.

Given the sampling methods used during this study, exhaustive screening among a large population fraction, our findings provide useful insight into the process of confirming yaws cases in areas of unknown distribution. In light of the extensive screening, the low observed prevalence and highly focal nature of the disease were particularly striking, reinforcing the limited feasibility of stand-alone survey-based approaches for detecting yaws. Surveys and case-finding activities for yaws should instead be considered when integrated alongside those for other skin NTDs to maximize efficiency (*12*,*27*). Our findings also support the use of larger implementation units for MDA at the county level, equivalent to the WHO definition of districts, following confirmation of an active yaws case, given the evident difficulty in excluding occurrence within smaller implementation units. We also sought only cases with active clinical symptoms of yaws disease, which means that our estimate of yaws infection is likely a substantial underestimate, given that the ratio of active to latent infection can be as high as 1:6 (*28*).

Although standalone surveys for yaws may be unsuitable, our results suggest that community-led case finding activities should be considered, including photo-based screening. Our accessibility analysis also provides some support for WHO guidance of purposively selecting remote communities for these activities (*11*). Our data suggested that the proportion of communities with yaws cases was greater among those classified as low or very low access. Our approach identified a single very-low-access cluster with high prevalence of confirmed yaws, thus supporting the idea that extremely remote settings may be at highest risk. The need for targeted case finding in areas of unknown distribution was further emphasized by the large sampling effort before identification of the first case in our survey process. Of note, however, half of all yaws-endemic communities in Maryland were defined as high access. Even though no cases were found in periurban or urban settings, these data show that if activities were focused solely on the most remote communities, most cases would be missed, a crucial point in light of eradication goals.

A recent modeling analysis indicated that, on the basis of several epidemiologic and structural factors, Liberia was unlikely to report yaws cases, even if present (*4*). Our findings indicate that knowledge and healthcare seeking for yaws among both healthcare workers and communities appeared low. Coupled with both our epidemiologic and accessibility findings, this contextual information highlights the challenges of yaws surveillance. We did, however, demonstrate that nonphysician healthcare workers could be trained to provide reliable clinical diagnoses of suspicious lesions despite limited prior awareness. These results are promising, given that the PPV and NPV of yaws can be low because of differential diagnoses (*29*,*30*). The high PPV attributable to papillomatous lesions also highlights that a clinical case definition of a yaws-like papilloma may be an effective tool in settings where serologic or molecular diagnostics are not available.

Our study’s limitations include the potential for missed yaws infections or cases of active disease. We did not screen every community in Maryland County, and community healthcare workers may have missed cases during case finding, nor did we train community healthcare workers to screen for the less common clinical manifestations of yaws. To reduce selection and classification bias, we administered rigorous training, real-time data monitoring, and quality control surveys in all community healthcare worker–surveyed clusters. Despite these efforts, both the prevalence of active yaws and genomic diversity of TPE in Maryland County could be greater than we report. In addition, we did not characterize latent yaws through widespread serologic testing; therefore, we cannot provide indications of the prevalence of latent infection. The inherent limitations of high host and bacterial contamination, combined with low *Treponema* load in swab specimens, permitted us to recover genomes from only 12 of 17 PCR-confirmed cases, meaning that we may have missed unsampled diversity.

Our use of exhaustive, rigorously validated sampling methods provides an unusual level of insight into the epidemiology of yaws and the public health challenges associated with confirming cases in areas of unknown burden. Coupled with the genomic characterization of TPE, we provide key details of TPE diversity in this region, which expands and reinforces understanding of TPE spatiotemporal diversity in West Africa. Other previously yaws-endemic countries can use our approaches and findings to inform surveillance activities and to support global yaws eradication efforts.

Appendix 1 Additional information about the methods involved in the epidemiologic and genomic reidentification of yaws, Liberia.

Appendix 2 Supplementary metadata about the epidemiologic and genomic reidentification of yaws, Liberia.
