# Supplementary material for: Epidemiologic and Genomic Reidentification of Yaws, Liberia
Source: Emerg Infect Dis. 2021 Apr;27(4):1123–32. doi: 10.3201/eid2704.204442 (PMC8007311; doi:10.3201/eid2704.204442)
Supplement: Appendix 1 — Additional information about the methods involved in the epidemiologic and genomic reidentification of yaws, Liberia. [file 20-4442-Techapp-s1.pdf]

Article DOI: https://doi.org/10.3201/eid2704.204442

Epidemiologic and Genomic Reidentification of Yaws, Liberia

Appendix 1

Methods

Survey Design

Maryland County (2018 estimated census population 165,456) is a largely rural and peri-urban county with Liberia's highest levels of absolute poverty (84.0% of population) (*1*). Before we began the survey activities, we analyzed routine surveillance data and we identified no cases of clinically suspicious or confirmed yaws reported in Maryland County. We conducted an integrated skin neglected tropical disease (NTD) burden estimation using a population-based cross-sectional survey design during June–October 2018. The survey had yaws as a primary outcome and Buruli ulcer, leprosy, and lymphatic filariasis morbidity as co-primary outcomes. All communities were eligible for inclusion; community health worker catchment areas were selected as primary sampling units. Contiguous community health worker catchments <300 persons were combined and those >1,000 divided before selection. In total, 92 clusters (arithmetic mean population = 618) stratified across all 24 health facilities were systematically selected using probability proportional to size with replacement. All residents of selected clusters were eligible and sought for participation in initial screening.

Procedures

Community health workers visited all households in selected clusters over a 7-day period and completed a simple census. During household visits, community health workers screened residents for skin NTDs using photos of clinical presentations. If household members were absent, the head of household or primary caregiver was asked to be a proxy respondent. Clinically suspected yaws cases were defined as those that any person verbally reported as exhibiting skin changes similar to the clinical photographs of yaws ulcers or papillomas. During a 3-day program, all community health workers were trained in the use of electronic data

collection tools running Open Data Kit (ODK)-based surveys (https://getodk.org). During household surveys, all community health workers collected GPS coordinates to validate coverage and allow extraction of GIS-based covariates. To ensure high community coverage, household GPS coordinates were overlaid onto satellite imagery during data collection activities and community health workers were informed of any missing areas for subsequent completion. We also conducted quality control surveys in all clusters following completion of community health worker activities. Trained healthcare workers visited a subpopulation of randomly selected households (14.0% of survey sample) to collect information on community health worker performance and to assess coverage through capture and recapture of QR-coded identify cards distributed by community health workers.

Following exhaustive community screening, we provided a case list to verification teams for home-based follow up of suspected cases. A clinically trained team member performed detailed examination and provided an initial clinical diagnosis. All persons with clinically suspicious cases of yaws or ulcers of alternative etiology underwent immediate testing for *Treponema pallidum* subspecies *pertenue* (TPE) antibodies following World Health Organization (WHO)-recommended procedures. Persons with clinically diagnosed cases were first tested for the presence of treponemal antibody using an SD Bioline syphilis lateral flow assay (https://www.globalpointofcare.abbott) followed by a ChemBio syphilis dual path platform (https://chembio.com) if positive. We collected 2 swabs from each lesion undergoing serologic testing; teams were trained to collect material from both the center and edge of lesions. Swabs were immediately placed into cell lysis solution (QIAGEN, https://www.qiagen.com) in chilled vaccine carriers. Samples were stored in the nearest health facility refrigerator before transport to a central −20°C freezer at JJ Dossen Hospital (Harper, Liberia). At the end of survey activities, we shipped the samples to London School of Hygiene and Tropical Medicine (UK) and the Wellcome Trust Sanger Institute (Hinxton, UK).

Before beginning survey activities, our team of 7 midlevel health workers (physician assistants) attended a 5-day training course on the diagnosis and management of skin NTDs led by the Ministry of Health NTD program and UK-based experts (M.M., S.L.W., J.T.), including a consultant tropical dermatologist. Training included tailored modules on the clinical diagnosis of yaws, differential diagnoses of yaws, and the use of yaws point-of-care tests. Training also included modules on all primary outcome skin NTDs and common skin infections. Knowledge

assessment of trainees was conducted using multiple-choice paper-based questionnaires to assesses pretraining awareness of the diagnosis and management of yaws and other skin diseases.

Community-Level Accessibility Data

To determine the characteristics of all survey communities, we extracted GIS-based information from all household coordinates collected by community health workers during screening (n = 9,375). Following current WHO guidelines prioritizing case finding in hard-to-reach communities, we extracted different open-source and custom-made data associated with accessibility and improved water coverage indicators, as follows:

- Population density (2018 UN-adjusted estimates; www.worldpop.org)
- Distance to Open Street Map roads (2016; www.worldpop.org)
- Distance to Open Street Map major road intersections (2016; www.worldpop.org)
- Distance to nearest city (2015; malariaAtlas version 1.0.1, www.malariaatlas.org)
- Improved housing coverage (2015; malariaAtlas version 1.0.1, www.malariaatlas.org)
- Land cover type (2016; www.worldpop.org)
- Euclidean distance to VIIR stable night lights (2012–2016; www.worldpop.org)
- Estimated travel time to health facility (AccessMod, https://www.accessmod.org/)
- Coverage of improved water sources (2017; Institute of Health Metrics and Evaluation, http://ghdx.healthdata.org/record/ihme-data/lmic-wash-access-geospatial-estimates-2000-2017)
- Coverage of improved sanitation (2017: Institute of Health Metrics and Evaluation), http://ghdx.healthdata.org/record/ihme-data/lmic-wash-access-geospatial-estimates-2000-2017

Sample Size and Statistical Analysis

We performed all data management and statistical analyses using R version 4.0.1 (https://www.r-project.org). Assuming a population-level prevalence of all skin NTDs of 5 per 10,000 population, absolute precision of 3.5 per 10,000, design effect of 3.5, and a participation rate of 0.8 and applying a finite population correction factor, the required sample size for county-

level prevalence was 48,478 using standard formulae. Prevalence estimates were made through design-based inference as a stratified one-stage cluster design with variance estimated using jackknife repeated replication (survey version 3.36). Intraclass correlation coefficients (ICCs) were estimated from intercept-only binomial mixed effects models (lme4 version 1.1–23; https://cran.r-project.org).

Whole Genome Sequencing

We screened all samples by quantitative PCR (qPCR), as previously described, to determine within-sample *Treponema* load (*2*,*3*). We performed whole genome sequencing directly on DNA extracted from clinical swabs, in parallel with *Treponema* samples from other studies, and grouped them with samples of similar bacterial load (qPCR Ct) in pools of 32. We prepared sequencing libraries using the pooled sequence-capture method previously described and using unique dual index barcoding (*2*,*4*). We sequenced pools of 32 samples/lane on Illumina HiSeq 4000 (https://www.illumina.com) to obtain 150 bp paired end reads.

Genome Analysis

Raw sequence-capture enriched sequencing reads were prefiltered, trimmed and downsampled to 2,500,000 *Treponema* reads, as previously described (*2*), using the full (dust-masked) bacterial and human Kraken2 database (March 2019) (*5*). We contextualized our Liberia genomes with 33 publicly available *T. pallidum* subspecies *pertenue* genomes from around the world, selected based on geographic distribution and genome coverage (minimum 84% breadth of genome coverage >5×), downloaded raw sequencing reads from the European Nucleotide Archive (https://www.ebi.ac.uk/ena/browser/home), and subjected them to the same binning and downsampling pipeline. Raw sequencing reads were unavailable for 5 public genomes, so we simulated 125 bp paired end perfect reads from the RefSeq genomes using fastaq (https://github.com/sanger-pathogens/Fastaq) as previously described (*2*). We included the BosniaA genome (*T. pallidum* subspecies *endemicum*, GenBank accession no. NZ_CP007548.1) as an outgroup.

For phylogenetic analysis, we used a custom version of the Samoa D reference genome (GenBank accession no. NC_016842.1), after first masking 14 highly repetitive or recombinogenic genes (12 repetitive Tpr genes A-L, arp and TPESAMD_0470/ tp0470) using bedtools v2.17.0 maskfasta (https://bedtools.readthedocs.io/en/latest). Filtered sequencing reads

were mapped to the reference using BWA mem v0.7.17 (MapQ ≥20; http://bio-bwa.sourceforge.net), followed by indel realignment using GATK v3.4–46 IndelRealigner (https://gatk.broadinstitute.org), deduplication with Picard MarkDuplicates v1.127 (http://broadinstitute.github.io/picard), and variant calling and consensus pseudosequence generation using samtools v1.68 and bcftools v1.6 (*6*), requiring ≥2 supporting reads per strand and 5 in total to call a variant, and a variant frequency/mapping quality cutoff of 0.8; sites not meeting these criteria were masked to "N" in the pseudosequence. After pseudosequence generation, we remasked the highly repetitive regions using remove_block_from_aln.py (https://github.com/sanger-pathogens/remove_blocks_from_aln). We screened our multiple sequence alignments for recombination using Gubbins v 2.4.1 (*7*), generating recombination-masked full-length genomes. We used snp-sites (*8*) to produce single nucleotide polymorphism-only alignments, and calculated maximum likelihood phylogenies using IQ-Tree (*9*) v1.6.10, inputting missing sites (inferred using snp-sites) using the "-fconst" argument, and specifying a general time reversible substitution model and FreeRate model of heterogeneity performing 1,000 UltraFast bootstraps (*10*).

We inferred macrolide resistance alleles using the competitive mapping method previously described (https://github.com/matbeale/Lihir_Treponema_2020). Trees were arranged in R using the ape v5.4–1 and phytools v0.7–47 packages. Phylogenetic and phylogeography figures were generating in R v3.6.0 using ggplot2 v3.3.2, ggtree v1.17.1, and ggmap v3.0.0. Maps used for phylogeography were downloaded from http://maps.stamen.com/ using the ggmap interface.

Ethics

The study protocol was approved by the University of Liberia (PIRE) Institutional Review Board (no. 18–02–088) and the Ethics Committee of the London School of Hygiene and Tropical Medicine (no. 14698). Community meetings were held in all study clusters before implementation. Verbal consent was obtained from adult residents for household participation in screening; written consent was obtained from residents, or guardians of persons under 18 years of age, for both quality control and case verification visits. All cases of active yaws and other skin conditions were immediately referred for treatment at health facilities in line with national guidelines. This study is registered with ClinicalTrials.gov, no. NCT03683745.

Results

Descriptive statistics of yaws and leprosy endemic communities from remote GIS datasets were extracted at cluster level for all survey communities. Variables were selected as a result of potential proxy measures of community accessibility (Appendix 1 Table 1).

Continuous data values (Appendix 1 Table 1) are cluster medians and interquartile range of all household-level GPS coordinates within each of the 92 survey clusters. Categorical variables are the most common value extracted within each cluster. Complete data were available for 9,375 of 10,007 survey point locations. Group comparisons were made using the Kruskal-Wallis or Fisher exact test.

Continuous data values (Appendix 1 Table 2) are cluster medians and interquartile range of all household-level GPS coordinates within each of the 92 survey clusters. Categorical variables are the most common value extracted within each cluster. Complete data were available for 9,375 of 10,007 survey point locations. Group comparisons were made using the Kruskal-Wallis or Fisher exact test.

References

1. Rogers JH, Jabateh L, Beste J, Wagenaar BH, McBain R, Palazuelos D, et al. Impact of community-based adherence support on treatment outcomes for tuberculosis, leprosy and HIV/AIDS-infected individuals in post-Ebola Liberia. Glob Health Action. 2018;11:1522150. PubMed https://doi.org/10.1080/16549716.2018.1522150

2. Beale MA, Marks M, Sahi SK, Tantalo LC, Nori AV, French P, et al. Genomic epidemiology of syphilis reveals independent emergence of macrolide resistance across multiple circulating lineages. Nat Commun. 2019;10:3255. PubMed https://doi.org/10.1038/s41467-019-11216-7

3. Beale M, Noguera-Julian M, Godornes C, Casadellà M, González-Beiras C, Parera M, et al. Yaws re-emergence and bacterial drug resistance selection after mass administration of azithromycin: a genomic epidemiology investigation. Lancet Microbe. 2020;1:e263–71. https://doi.org/10.1016/S2666-5247(20)30113-0

4. Marks M, Fookes M, Wagner J, Butcher R, Ghinai R, Sokana O, et al. Diagnostics for yaws eradication: insights from direct next-generation sequencing of cutaneous strains of *Treponema pallidum*. Clin Infect Dis. 2018;66:818–24. https://doi.org/10.1093/cid/cix892

5. Wood DE, Lu J, Langmead B. Improved metagenomic analysis with Kraken 2. Genome Biol. 2019;20:257. PubMed https://doi.org/10.1186/s13059-019-1891-0

6. Li H, Handsaker B, Wysoker A, Fennell T, Ruan J, Homer N, et al.; 1000 Genome Project Data Processing Subgroup. The Sequence Alignment/Map format and SAMtools. Bioinformatics. 2009;25:2078–9. PubMed https://doi.org/10.1093/bioinformatics/btp352

7. Croucher NJ, Page AJ, Connor TR, Delaney AJ, Keane JA, Bentley SD, et al. Rapid phylogenetic analysis of large samples of recombinant bacterial whole genome sequences using Gubbins. Nucleic Acids Res. 2015;43:e15. PubMed https://doi.org/10.1093/nar/gku1196

8. Page AJ, Taylor B, Delaney AJ, Soares J, Seemann T, Keane JA, et al. *SNP-sites*: rapid efficient extraction of SNPs from multi-FASTA alignments. Microb Genom. 2016;2:e000056. PubMed https://doi.org/10.1099/mgen.0.000056

9. Nguyen LT, Schmidt HA, von Haeseler A, Minh BQ. IQ-TREE: a fast and effective stochastic algorithm for estimating maximum-likelihood phylogenies. Mol Biol Evol. 2015;32:268–74. PubMed https://doi.org/10.1093/molbev/msu300

10. Hoang DT, Chernomor O, von Haeseler A, Minh BQ, Vinh LS. UFBoot2: Improving the ultrafast bootstrap approximation. Mol Biol Evol. 2018;35:518–22. PubMed https://doi.org/10.1093/molbev/msx281

Appendix 1 Table 1. Population accessibility indicators for all survey communities, stratified by presence of ≥1 yaws case*

Indicator	Yaws absent	Yaws endemic	p value
Total communities	84	8	
High access	61 (72.6%)	4 (50.0%)	0.04
Low access	23 (27.4%)	3 (37.5%)	
Very low access	0	1 (12.5%)	
Rural	72 (85.7%)	8 (100%)	0.59
Peri-urban or urban	12 (14.3%)	0	
Population density/km^2	116.0 (79.2–234.9)	61.43 (55.2–84.1)	0.01
Travel time to health facility (minutes)	49.0 (19.5–104.2)	68.00 (22.7–184.0)	0.42
Travel time to nearest city (minutes)	170.0 (151.2–196.5)	193.0 (161.7–324.0)	0.08
Distance to stable night lights (km)	7.7 (3.5–15.5)	12.2 (8.3–15.3)	0.22
Distance to OSM road intersection (km)	6.5 (1.0–11.4)	8.3 (4.6–10.3)	0.35
Distance to OSM road (km)	0.38 (0.09–3.06)	3.38 (0.65–6.66)	0.03
Coverage of improved water	0.94 (0.79–0.98)	0.96 (0.84– 0.99)	0.53
Coverage of improved sanitation	0.23 (0.10–0.37)	0.32 (0.25–0.37)	0.25
Coverage of improved housing	0.10 (0.06–0.13)	0.07 (0.06– 0.10)	0.09

*Values with intervals indicate median (interquartile range).

Appendix 1 Table 2. Population accessibility indicators for all survey communities, stratified by presence of ≥1 leprosy case*

Indicator	Leprosy absent	Leprosy endemic	p value
Total communities	65	27	
High access	43 (66.2%)	22 (81.5%)	0.38
Low access	21 (32.3%)	5 (18.5%)	
Very low access	1 (1.5)	0	
Rural	55 (84.6%)	25 (92.6%)	0.50
Peri-urban or urban	10 (15.4%)	2 (7.4%)	
Population density/km^2	109.5 (71.2–258.9)	108.5 (87.8–148.5)	0.91
Travel time to health facility (minutes)	56.0 (21.0−118.0)	47.0 (18.0–99.50)	0.41
Travel time to nearest city (minutes)	170.0 (152.0–209.5)	170.0 (153.5–192.5)	0.61
Distance to stable night lights (km)	7.1 (2.2–15.0)	11.2 (5.2–16.8)	0.25
Distance to OSM road intersection (km)	7.0 (1.2–11.5)	6.5 (2.1–11.0)	0.92
Distance to OSM road (km)	0.65 (0.09–3.43)	0.21 (0.09–2.56)	0.28
Coverage of improved water	0.94 (0.78–0.98)	0.95 (0.84–0.98)	0.97
Coverage of improved sanitation	0.24 (0.11–0.37)	0.26 (0.13–0.37)	0.84
Coverage of improved housing	0.11 (0.06–0.13)	0.08 (0.07–0.12)	0.80

*Values with intervals indicate median (interquartile range).
